# Assessing base rates of sexual behavior using the unmatched count technique

**DOI:** 10.1080/21642850.2014.886957

**Published:** 2014-02-20

**Authors:** Amy J. Starosta, Mitch Earleywine

**Affiliations:** ^a^ Clinical Psychology, University at Albany, 1400 Washington Blvd, Albany, NY12222, USA

**Keywords:** HIV/AIDS, sexual and reproductive health, mixed-methods research

## Abstract

Estimating the prevalence of sexual behaviors is difficult because of self-report biases. This is particularly relevant in assessing high-risk sexual behaviors for the purpose of reducing the transmission and acquisition of sexually transmitted infections (STIs) and HIV/AIDS. The present study employed the unmatched count technique (UCT), which provides estimates of the prevalence of risky sexual behaviors without requiring participants to confess to socially undesirable or stigmatized behaviors. Compared to a standard, anonymous self-report questionnaire, the UCT protocol revealed that people were less likely to notify their partners about STIs or discuss their history of sexual experiences. Effects were particularly large in women suggesting that women may be more likely to misrepresent their sexual behaviors. The findings suggest that conventional, anonymous self-report questionnaire data of base rates of risky sexual behavior and sexual communication are consistently inaccurate. These discrepant base rates suggest that the UCT might provide a better estimate of the frequency of these behaviors. Results suggest that inconsistent sexual behavior is more rampant than anonymous questionnaires suggest. They also underscore the need for improvements in the anonymity of assessment of sexual behaviors, which could in turn improve the targeting of prevention efforts. Results have important public health implications because accurate assessment of sexual behaviors is crucial for developing effective STI prevention interventions among target populations.

## Introduction

Current research on sexual behavior relies heavily on self-reports (Cleland, Boerma, Carael, & Weir, 2004; Graham, Crosby, Sanders, & Yarber, 2005; Obermeyer, 2005; Schroder, Carey, & Vanable, 2003). However, accurately assessing sexual behavior is extremely difficult due to a variety of factors. For many, sexual behavior is a very private activity, and public discussion of sexual behaviors may have negative connotations. Further complicating the issue, social desirability, varied interpretations of questions, and problems in recall can impede accurate measurements of risky sexual behavior (Brewer, Garrett, & Kulasingam, 1999; Brody, 1995; Schroder et al., 2003). These barriers are not unique to the measurement of sexual behaviors. In fact, all self-report surveys can be susceptible to both under- and over-reporting (Rosenthal & Rosnow, 2008). Misreporting on sensitive topics is very common; participants frequently edit responses to avoid embarrassment or repercussions (Tourangeau & Yan, 2007). For example, in a study assessing the impact of anonymous assessment on reporting of substance abuse in an HIV+ population, researchers found that anonymous surveys yielded significantly higher rates of substance abuse reporting than confidential surveys (Hormes, Gerhardstein, & Griffin, 2012).

It is critical to develop accurate tools to assess sexual behavior, particularly in the context of risky sexual behaviors that can lead to the transmission and acquisition of sexually transmitted infections (STIs). STIs have approached epidemic status in the USA and disproportionately affect young and minority populations (American College Health Association, 2008; Centers for Disease Control and Prevention [CDC], 2011a, 2011b; Weinstock, Berman, & Cates, 2004). STIs cause significant medical complications, such as infertility and, in extreme cases, death. Not only do STIs have medical consequences, they also have financial cost for society as a whole. Negative consequences from STIs cause direct medical costs of nearly $15.5 billion per year (Chesson, Blandford, Gift, Tao, & Irwin, 2004). Given their prevalence and costs, the need for interventions to decrease the incidence of STIs is paramount.

In an attempt to increase the efficiency of interventions to reduce STIs, the US CDC now advocates that all interventions should target high-risk populations. However, in order to target high-risk populations, researchers must first identify those groups that are engaging in high-risk behaviors. This can be difficult as identifying those at risk requires assessment of risky sexual behaviors that many perceive as embarrassing, intrusive, or inappropriate (Janssen et al., 2001). Targeting interventions based on inaccurate assessments of risky sexual behaviors can potentially result in misguided or ineffective interventions. Accurate reporting can be increased through techniques such as self-administration or increased anonymity (Tourangeau, Rasinski, Jobe, Smith, & Pratt, 1997; Tourangeau & Smith, 1996). Given the various perspectives, emotions, and potential repercussions, one critical component in developing effective assessment for sensitive behaviors is anonymity. An alternative approach with increased anonymity might provide more accurate estimates of relevant behaviors and improve efforts at targeted prevention.

### The unmatched count technique

One method of assessing behaviors in an anonymous fashion is the unmatched count technique (UCT). The UCT is a means to assess the prevalence of behaviors within a specific population or demographic without requiring individual participants to endorse specific undesirable acts (Wimbush & Dalton, 1997). Because participants never endorse one specific behavior, it is impossible to ascertain which behaviors are true for each individual, therefore providing greater actual and perceived anonymity. Rather, the UCT provides a tool for estimating the base rate of behaviors within a group. Therefore, it provides an opportunity for accurate assessment of risky sexual behavior within specific populations.

In the UCT, participants receive a set of statements, wherein they indicate the number of statements that are true for them, without specifying which ones. One group receives a set of innocuous items (e.g. five statements about common demographics or preferences). A second group receives those same five items plus a target, sensitive item (e.g. something relevant to sexual behaviors). The base rate is derived by computing the difference between the average endorsement level for the group with and without the target item. Given large samples and random assignment, this estimate should reveal the percentage of people who engaged in the target, sensitive behavior.

For example, if the group without the target item endorses an average of 2 statements and the group with the target item endorses an average of 2.2 statements, then the difference between the rates of endorsement is 0.20. This finding suggests that 20% of people endorse the sensitive item when it is presented, and thus suggests a base rate of 20% for the target behavior. Previous work employing this method revealed higher base rates for employee theft, disordered eating, hate crime victimization, and condom and alcohol use (Anderson, Simmons, Milnes, & Earleywine, 2007; LaBrie & Earleywine, 2000; Rayburn, Earleywine & Davison, 2003; Wimbush & Dalton, 1997). Sexual behavior is a very sensitive topic and the accurate assessment of sexual behavior is critically important given the current public health implication. The UCT protocol might be an ideal method to assess base rates of risky sexual behavior.

### Present study

We built on a prior study that employed the UCT method to examine reporting of sex without a condom (LaBrie & Earleywine, 2000). In previous work, the UCT method revealed that 70% of participants reported having had sex without a condom, but the conventional, anonymous self-report questionnaire suggested that only 59% had done so, a discrepancy of 11%. In addition, the UCT revealed that 65% of participants had sex without a condom after alcohol consumption while the conventional, anonymous self-report questionnaire suggested that only 36% had done so, a discrepancy of 29%. We extended this line of questioning to other, particularly risky, sexual behaviors.

Because the previous data were gathered over a decade ago, standards and norms for sexual behaviors may have changed, suggesting the need for re-evaluation. We also aimed to extend previous findings by focusing on more interpersonal aspects of safe sex. The three target items used in the current study were: “I ask my partner about their sexual history before engaging in sexual intercourse,” “I ’ve had sex without a condom,” and “If I had a STD that was actively being treated, I would alert my current partner.” (While STI is the accepted nomenclature in the field, among the general population, sexually transmitted disease (STD) is more commonly recognized. In the interest of clarity, we used the more common phrase for the research study, but for the purpose of this paper, will use STI.) We predicted that the UCT responses would reveal higher endorsement rates of sensitive behaviors such as having sex without a condom and lower endorsements of behaviors such as alerting a partner of an STI or asking about a partner's sexual history.

Assuming that self-reported base rates of high-risk sexual behavior are inaccurate estimates because responders are concerned with anonymity (among other related issues), percentages of endorsements derived using the UCT protocol will provide a more accurate representation of risky sexual behavior. These findings may help public health officials assess risk within a given population to target interventions to those most in need. Additionally, base rates can be used as a comparison point for future assessments of risky sexual behavior. Additionally, assessment tools with increased anonymity may decrease discomfort for respondents.

## Methods

### Participants

Participants were recruited via Internet posting on sites like Craig's List and Facebook as well through the psychology research pool of a Northeastern university. One thousand two hundred and ninety-one participants entered the online survey management site used to administer the questionnaires. Five participants did not consent to the terms of the study and did not complete the questionnaires. Because the study was administered on a public website and the procedures were conducted in private without any supervision from the researchers, we elected to insert an infrequency item to eliminate any participants who were not actively engaged in the questionnaires. Infrequency items are items which will elicit the same response from all attentive respondents. For the present study, we included an item stating “Please select ‘strongly disagree’ in concurrently administered survey.” A failure to endorse “strongly disagree” on this item was assumed to reflect insufficient effort or attention on the part of the respondent. Two hundred and thirty-one participants were removed from analysis for incorrectly responding to the infrequency item. Given the constraints of the participant recruitment in this study, it is not surprising to have a high number of participants who did not fully engage in the questionnaires.

### Procedures

All questionnaires were administered via an online survey management site (surveymonkey.com), and all procedures were reviewed and approved by the local university Institutional Review Board. Participants provided informed consent and answered basic demographic information such as age, race, and gender in addition to sexual history questions such as relationship status, sexual preference, and previous condom use. Participants were not asked any identifying questions, such as name or email. Additionally, participants filled out a survey unrelated to the current study. After demographic questionnaires were completed, participants were randomized to one of three conditions based on the day of their birth. Participants born on days numbered 1–10 were in Group 1, participants born on days numbered 11–20 were in Group 2, and those born on days numbered 21–31 were in Group 3. There were no significant differences between groups on any demographic or sexual behavior measures (Table 1). There were some slight differences in sample sizes (*N* = 318, 381, and 317). However, all analyses are based on the average response rate of each group. Given that using the average takes into account sample size, Wimbush and Dalton's (1997) UCT study reported that with sufficient sample size and random assignment, moderate differences in sample size should not impact outcomes.

**Table 1.** Group differences.

Demographics	Group 1, *N* = 319	Group 2, *N* = 381	Group 3, *N* = 317	Total, *N* = 1055	*p* Value
Age, mean (SD)	24.50 (6.47)	24.85 (8.09)	24.15 (6.96)	24.52 (7.26)	0.860
*Gender, no. (%)*					
Male	112 (35.1)	134 (35.2)	119 (37.5)	365 (35.9)	0.761
Female	207 (64.9)	247 (64.8)	198 (62.5)	652 (64.1)	
*Caucasian, no. (%)*					
Yes	255 (79.9)	287 (74.7)	240 (75.7)	782 (76.7)	0.238
No	64 (20.1)	97 (25.3)	77 (24.3)	238 (23.3)	
*African-American, no. (%)*					
Yes	19 (6.0)	36 (9.4)	27 (8.5)	82 (8)	0.235
No	300 (94)	348 (90.6)	290 (91.5)	938 (92)	
*Hispanic/Latino, no. (%)*					
Yes	19 (6.0)	30 (7.8)	20 (6.3)	69 (6.8)	0.576
No	300 (94)	354 (92.2)	297 (93.7)	951 (93.2)	
*Asian, no. (%)*					
Yes	22 (6.9)	32 (8.3)	25 (7.9)	79 (7.7)	0.772
No	297 (93.1)	352 (91.7)	292 (92.1)	941 (92.3)	
*Other ethnicity, no. (%)*					
Yes	14 (4.4)	17 (4.4)	14 (4.4)	45 (4.4)	1.000
No	305 (95.6)	367 (95.6)	303 (95.6)	975 (95.6)	
*Sexually active, no. (%)*					
Yes	291 (91.2)	355 (92.4)	286 (90.8)	932 (91.6)	0.713
No	28 (8.8)	29 (7.6)	29 (9.2)	86 (8.4)	
Number of sexual partners in the past year, mean (SD)	2.85 (6.1)	2.76 (10.5)	2.53 (3.9)	2.72 (7.6)	0.067
*Primary gender of sexual partners, no. (%)*					
Opposite sex	286 (90.2)	346 (92.0)	288 (90.9)	920 (91.1)	0.698
Same sex	31 (9.8)	30 (8.0)	29 (9.1)	90 (8.9)	
*Condom use at last sex act, no. (%)*					
Yes	171 (53.8)	231 (60.5)	186 (58.7)	588 (57.8)	0.328
No	147 (46.2)	151 (39.5)	131 (41.3)	429 (42.2)	
*Relationship status, no. (%)*					
Monogomous	124 (39.6)	146 (38.6)	136 (44)	406 (40.6)	0.698
Single	189 (60.4)	232 (61.4)	173 (56)	594 (59.4)	
Length of relationship (months), mean (SD)	30.06 (44.2)	23.82 (39.7)	21.92 (30.5)	25.02 (38.6)	0.458

Note: Group differences assessed using analysis of variance and χ^2^ tests.

#### Conventional, anonymous self-report questionnaire protocol

Group 1 received a series of behavioral statements, including the sensitive questions of interest. They were informed that all answers would be anonymous and were asked to answer if each statement was true or false for them. This group simulates the traditional assessments of sexual risk taking, and we hypothesize that this method will yield inaccurate estimates of sexual behaviors. All items used in this protocol appear in Appendix 1.

#### UCT protocol

Base rate estimates by UCT require two randomly assigned groups (Wimbush & Dalton, 1997). One group answers a set of five non-sensitive questions, while the second group responds to these five statements plus an extra item. This extra item is the item of interest. Participants in both groups are asked not to respond to any specific item, but rather to indicate how many of the items in the list are true for them. Group 2 had two sets with extra items and one without an extra item. Group 3 had one set with an extra item and two sets without an extra item (Appendix 2). These two groups were yoked together so that Group 2 answered the sets without the sensitive items for Group 3, and Group 3 answered the sets without the sensitive items for Group 2.

As participants are randomly assigned and there were no significant group differences, any difference in the mean endorsement of these two sets of questions can theoretically be attributed to participants in the second group endorsing the sensitive item. The base rate for the sensitive behavior is determined from the difference between the mean endorsement of the first group and the mean endorsement of the second group. An example item reads:

I consider myself a sports fan.

I live in the same state as my parents.

I often make “to-do” lists.

I've had a monogamous relationship.

I enjoy scrapbooking.

If a participant gave a response of 2 to this set, then two of the five items are true for this participant. The computerized survey did not allow answers to individual items; participants could only indicate the total number of the set that was true for them. The UCT group received the exact same set of statements, but with the additional statement “I ask my sexual partners about their sexual history.” For example, the 310 participants who answered the control question set endorsed a mean of 3.08 of the five items. The 379 participants who answered the question set containing the sensitive item endorsed a mean of 3.69 of the six items. The second group is expected have a higher mean endorsement rate, as there is an additional item. As participants have been randomly assigned, the difference between these two groups is a function of those participants who endorsed the additional item, asking sexual partners about their sexual history (Wimbush & Dalton, 1997).

The base rate for the behavior of interest is determined by subtracting the two means: *p* *=* *M*_UCT_ *− M*_Control_, where *p* is the proportion of participants endorsing the sensitive behavior. In this example, *p* = 3.69−3.08 = 0.61. Therefore, the percentage of participants in this population that endorse the statement “I ask my sexual partners about their sexual history” is 61%.

Large sample size and random assignment minimize the chance that confounds rather than participant endorsement of the sensitive behavior accounts for the difference between the two groups. Dalton and Wimbush (1994) suggest that in order to maintain accuracy and stability, groups should contain at least 40–50 participants. In this study, the smallest group had 57 participants (single males, UCT Group 3); however, the average number of participants per group was 160.

A traditional benchmark for the effectiveness of UCT is whether it results in significantly different rates of endorsement of undesirable behaviors than more conventional self-report techniques (Dalton & Wimbush, 1994; Wimbush & Dalton, 1997). The UCT could reveal higher rates for the “I've had sex without a condom” item. However, base rates could also be over-estimated by conventional self-report surveys, especially when the sensitive behavior is something that is pro-social but difficult or embarrassing, as in the case of the items “If I had an STD that was actively being treated, I would alert my current partner,” and “I ask my partner about their sexual history.” For each of these statements, UCT should result in lower base rates than conventional self-report.

### Statistics

Statistical analyses were modeled based on the Wimbush and Dalton UCT study (1997). Because each participant either engaged in the behavior or did not, the data are suited for analysis using independent binomials. The null hypothesis is that the proportion of endorsements in the conventional self-report and the proportion of endorsement in the UCT protocol are equal. All descriptive analyses of group endorsement rates were conducted using SPSS 17.0. Endorsement rates were calculated using the procedure described above. Finally, *Z*-scores and significance levels of the differences in endorsement rates (or proportions) were then calculated. Because of multiple questions and concerns about increased error rates, we adopted a conservative alpha of *p* < .001 for each comparison to indicate significant differences.

## Results

### Participants

In total, 1055 participants were included in the final analysis (379 men (36%), 673 women (64%), three declined to indicate gender, *M*_age_ *__*= 24.61 years, SD* *= 7.38, age range: 18–65). Ethnically, 77.1% of participants identified as Caucasian, 7.9% as African-American, 7.7% as Asian, 6.6% as Hispanic/Latino, and 4.5% identified as Other. The cumulative total is larger than 100% as participants were able to identify as more than one race. Overall, 91.2% of respondents were sexually active, and 57.6% were currently in a monogamous relationship. Nine hundred and fifty-one (90.1%) of the respondents reported primarily engaging in heterosexual behavior while 56 males (14.8%) and 36 females (5.4%) reported engaging in primarily gay or lesbian sexual behavior.

Although it may not seem immediately applicable to ask participants that self-identify as lesbian about condom use or participants that are not sexually active about sexual behavior, we included these populations in the analysis. Questions about sexual history discussions and STI notification are still applicable for the lesbian community. Additionally, while some participants may not be currently sexually active, they still can have expectations about condom provisions, sexual history discussions, and STI reporting, and these expectations provide insight to future sexual behaviors.

### Findings

The first set of comparisons was performed using all participants (*N* = 1015; Table 2). There were significant differences in endorsements of the statements “I've had sex without a condom” (*p *≤ .0002), “I ask my partner about their sexual history before engaging in sexual intercourse” (*p* ≤ .0001) and “If I had an STD that was actively being treated, I would alert my current partner” (*p *≤ .0001). As anticipated, a lower rate of endorsement of the sexual history discussion and STI notification items was observed in the UCT condition, suggesting that people tend to over-report sexual history discussions and STI notification. However, counter to our predictions, there was also a lower rate of endorsement in the UCT condition for the sex without a condom item, suggesting that in general, individuals actually tend to over-report sex without a condom. Examining responses provided by subsets of participants could reveal if some groups are more likely to alter responses in the UCT condition.

**Table 2.** Conventional anonymous self-report questionnaire and UCT results by group.

All participants	Conventional % (*n* = 326)	UCT % (n = 689)	*Z-*value^a^	Factor score^b^
Sex without a condom	86	76	3.666*	0.88
Sexual history	76	61	4.640*	0.81
STD notification	90	71	6.921*	0.78
Females only	Conventional (*n* = 210)	UCT % (*n* = 442)	*Z*-value	Factor score
Sex without a condom	86	74	3.510*	0.86
Sexual history	79	59	4.913*	0.75
STD notification	92	72	5.771*	0.78
Males only	Conventional (*n* = 116)	UCT % (*n* = 244)	*Z*-value	Factor score
Sex without a condom	86	79	1.638	0.92
Sexual history	71	64	1.256	0.91
STD notification	88	71	3.554*	0.81
Single total	Conventional (*n* = 149)	UCT % (*n* = 278)	*Z*-value	Factor score
Sex without a condom	83	68	3.243*	0.92
Sexual history	67	47	3.972*	0.91
STD notification	92	69	5.358*	0.81
Single female	Conventional (*n* = 88)	UCT % (*n* = 163)	*Z*-value	Factor score
Sex without a condom	82	69	2.192	0.84
Sexual history	70	45	3.867*	0.64
STD notification	93	54	6.339*	0.58
Single male	Conventional (*n* = 61)	UCT % (*n* = 115)	*Z*-value	Factor score
Sex without a condom	84	52	4.131*	0.62
Sexual history	62	43	2.437	0.69
STD notification	90	87	0.624	0.96

*Significance at *p* < .001 level.

^a^The *Z*-score provides the significance distribution for a difference of proportion test and indicates whether the difference in the percentages noted is statistically significant.

^b^The factor score is derived by dividing the UCT percentage by the conventional. The “0.88” for the “sex without a condom” level indicates that participants under the UCT approach are 0.88 times less likely to admit to sex without a condom than those responding under the conventional survey methods.

Although the subgroups are smaller than the total sample, restricting power, we still noted interesting differences between the UCT and conventional, anonymous self-report questionnaire conditions within these groups. Examining only females did not alter the findings (*N* = 652; Table 2); women showed the same pattern of differences as the total sample. Among men (*N* = 360), the only significant discrepancy was that men in the conventional condition reported lower rates of STI notification than those in the UCT condition; they did not show differences on the other items.

As sexual behaviors are often affected by relationship status (Macaluso, Demand, Artz, & Hook, 2000), we compared the responses of participants who were not currently in a monogamous relationship. While the smaller sample size of this population may limit any broad conclusions, preliminary analysis showed that there were also discrepancies between the conventional, anonymous self-report questionnaire and UCT conditions among single participants (*N* = 427). Those participants in the UCT condition reported lower rates of sex without a condom, sexual history discussion, and STI notification. There were also slight differences between the responses of single females and males. Single females in the UCT condition showed significantly lower rates of endorsement of sexual history discussion and STI notification, while single males in the UCT condition only showed significant lower reporting on the sex without a condom item (Table 2).

### Gender moderated disparities

**Figure 1.**
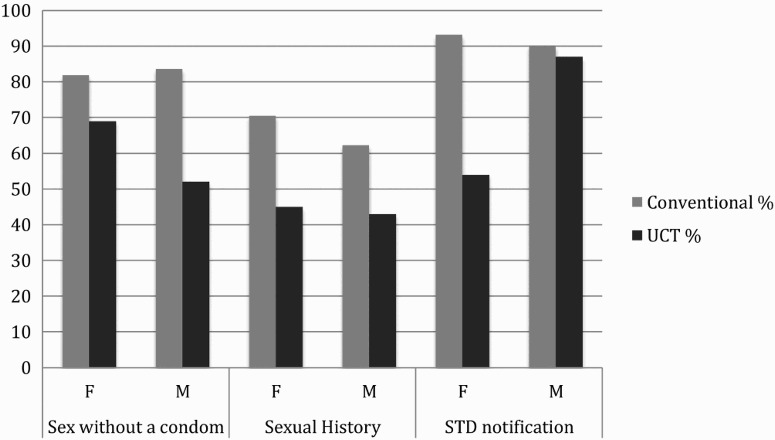
Female *vs.* male single participants.

Finally, we were interested in gender differences in the reporting of responsible sexual behaviors, as gender may influence perceptions of and attitudes toward responsible sexual behavior (Crosby et al., 2013). Using the separate analyses of the endorsement rates for men and for women, we assessed the difference between these two groups in the discrepancies between the UCT and conventional, anonymous self-report questionnaire conditions. Using the difference between proportions of behaviors endorsed in UCT and conventional, anonymous self-report questionnaire among participants in a relationship, women showed a significantly greater discrepancy than men in the sexual history discussion item (*z* = −7.09, *p *≤ .0001), suggesting that women in the conventional, anonymous self-report questionnaire condition evidenced greater over-reporting of sexual history discussions than men. Among single participants, men may be more likely to over-report sex without a condom as they showed a significantly greater discrepancy in the sex without a condom item (*z* = −4.27, *p *≤ .0001). Additionally, women showed significantly greater over-reporting on the STI notification item (*z* = 8.56, *p *≤ .0001) (Figure 1). Overall, women showed a greater tendency to over-report responsible sexual behaviors than men.

## Discussion

On the whole, the UCT condition revealed significantly lower base rates for sex without a condom, STI notification, and sexual history than a conventional, anonymous self-report questionnaire condition with an average of 17% points difference in the base rates estimated. Based on these findings, it appears that participants are over-reporting when it comes to sexual communication (i.e. STI notification and discussions about sexual history). However, the unexpected finding is that participants are over-reporting risky sexual behaviors (i.e. sex without a condom) as well. While this finding is surprising, false reporting of sexual risk behaviors could be associated with impression management. International research on the stigma surrounding condom use suggests that condom use is often associated with illicit sex or unclean partners (Buck et al., 2005; Roth, Krishnan, & Bunch, 2001; Wong & Yilin, 2003). Perhaps this stigma is also applicable in the USA, resulting in participants being less willing to admit to condom use as a result of the stigma associated with it. Further research is needed to examine the influence of stigma on condom use reporting.

Analyses of each gender separately revealed that women in the UCT condition showed significant lower rates of endorsement of sex without a condom, STI notification, and sexual history items, while men in the UCT condition showed significantly lower rates of endorsement in STI reporting only. Exploratory analyses with the subset of participants who were not in monogamous relationships suggested that these overall patterns are slightly different based on relationship status, as single women showed over-reporting of sexual history discussion and STI notification, while single men only showed over-reporting of sex without a condom. However, our sample contained a limited number of single males, precluding an extensive analysis of this group.

While the UCT condition revealed that over-reporting responsible behaviors was common, the discrepancy between the self-report and UCT condition was larger for women than for men. Not only were women over-reporting behaviors in more categories, the discrepancies between the UCT and conventional, anonymous self-report questionnaire conditions were larger for women than for men; Single women in the conventional, anonymous self-report questionnaire condition reported significantly higher rates of endorsement for STI notification and sexual history discussion, while men in the conventional, anonymous self-report questionnaire condition appear to only over-report having sex without a condom. Several factors may lead to different reporting by gender. Female participants could be interpreting the questions differently from the way that male participants interpret them. When discussing sexual history with a partner, men could be thinking about number of partners, while women may be more focused on past record of STIs. Even if women are thinking about number of partners, women have been found to be consistently less willing to report on numbers of non-marital sexual partners, which is likely an artifact of reporting biases (Brown & Sinclair, 1999; Cleland et al., 2004; Pedersen, Miller, Putcha-Bhagavatula, & Yang, 2002). Negative stigma associated with sexual behavior for women could also lead to differing reports, as men may not feel the same negative evaluation for having multiple partners or for engaging in risky sexual behaviors (Alexander & Fisher, 2003). According to theories of a “sexual double standard,” men who have many sexual partners are rewarded with increased social status, while women with multiple partners are stigmatized and villainized (Conley, Ziegler, & Moors, 2011; Jonason, 2007; Jonason & Fisher, 2009). In the same vein, men who embody societal masculine stereotypes may be more likely to report risky sexual behavior to preserve “masculinity” (Shearer, Hosterman, Gillen, & Lefkowitz, 2005). These findings emphasize the importance of clear and accurate assessment of sexual behaviors, especially for female populations, as self-report surveys seem to be providing inaccurate base rates of behavior. The discrepancies among reporting for women suggests that educational and intervention services should seek to decrease the stigma surrounding sexual activity in order to promote more open and accurate reporting. Additionally, as generality in the question items may have led to gender-specific interpretation, future research using UCT should focus on more specific behaviors.

The present study is limited by several important considerations. First, the participants were a self-selected subset of the population; all participants were internet savvy, familiar with community websites, and willing to answer questions about their sexual behavior. Bearing this in mind, caution should be used in generalizing the result from this study to other populations. However, the non-random sample does not detract from the UCT as an assessment tool, and future use of the UCT in more specific populations may provide information regarding the base rates of different risky behaviors within those populations. Additionally, the sample was comprised more female than male participants. Given that the results of the study suggest a larger discrepancy in reporting among women, it is possible that this finding is an artifact of a larger sample of women. Furthermore, there is a relatively small sample of single participants. However, since the overall sample well exceeds the suggested criteria established by Dalton and Wimbush (1994), it is unlikely that the gender moderated discrepancies are due to the differences in sample sized. Unfortunately, specific analysis of participants not in a monogamous relationship was hampered by a lack of single males. Additionally, our sample was not ethnically diverse. Base rates of risky sexual behavior are particularly important to assess within an African-American population as African-Americans are disproportionally affected by STIs and HIV/AIDS (Jemmott, Jemmott, & O'Leary, 2007). Single and minority populations are likely at higher risk of STIs, and therefore assessment of the base rates of risky sexual behavior in single and minority populations is critical for future research. While we assessed factors such as race, gender, age, and relationship status, previous research has found that other factors, such as personality (Bancroft et al., 2005), could affect sexual behaviors. Future studies examining sexual risk behavior using UCT might include religious affiliations and sensation seeking measures when checking for group equivalence.

Finally, while UCT allows for more anonymous estimation of base rates, the nature of the method does not allow researchers to link other data to individuals or to identify which individuals do or do not engage in risky sexual behaviors. Since it is impossible to know which behaviors are endorsed, it is also impossible to link certain behaviors to other traits. However, the potentially more accurate base rates revealed by UCT may be helpful in adjusting the interpretation of other findings that employ more conventional methodologies.

Accurate assessment tools for the prevalence of risky sexual behaviors are critical for research on factors that lead to risky sexual behaviors. Precise assessment is also imperative in the development of educational and intervention services to reduce risky behaviors. The present study suggests that conventional, anonymous self-report survey data of base rates of risky sexually behavior and sexual communication is likely inaccurate. The UCT protocol reveals lower base rates for sex without a condom as well as lower base rates for STI notification and sexual history discussion. Therefore, it appears that UCT is a superior assessment tool for determining base rates of these sensitive behaviors which may be incorrectly reported in self-report data. This protocol has the potential to be vital to future sex research and could be particularly useful in assessing things like disclosure of HIV status, sexual behavior among serodiscordant partners, or sharing of needles during intravenous drug use. There is likely significant misreporting of risky behaviors in current HIV and STI-related research as well as health and sexual behaviors more broadly speaking. Accurate base rates are essential to understanding associations between risky behavior and prevention of STIs and HIV/AIDS, and UCT is one technique that could assist in the development of more precise base rates of these behaviors.

## Appendix 1. Conventional, anonymous self-report survey items

Group 1

1. I drink 6–8 glasses of water a day
2. I like to cook.
3. I've had sex without a condom.
4. I sleep less than 7 hours per night.
5. I keep ticket stubs.
6. I've use Craig's list.
7. I consider myself a sports fan.
8. I live in the same state as my parents.
9. I often make “to-do” lists.
10. I've had a monogamous relationship.
11. I ask my partner about their sexual history.
12. I enjoy scrapbooking.
13. My parents were harsh with discipline.
14. I see my dentist every six months.
15. I regularly do Kegel exercises.
16. I am a loyal friend.
17. I don't mind when dishes pile up in the sink.
18. I floss everyday.
19. I have few photos of myself.
20. I don't procrastinate.
21. I get down in the dumps for a few days in the row sometimes.
22. If I had an STD that was actively being treated, I would alert my current partner.
23. I have stalked people on Facebook.

## Appendix 2. Unmatched count technique behavior sets

Instructions: for the next four questions, please answer how many of these statements are true for you.

Group 2

I drink 6–8 glasses of water a day.
I like to cook.
I've had sex without a condom.
I sleep less than 7 hours per night.
I keep ticket stubs.
I've used Craig's list.
How many of the above statements are true for you?
I consider myself a sports fan.
I live in the same state as my parents.
I often make “to-do” lists.
I've had a monogamous relationship.
I ask my partner about their sexual history.
I enjoy scrapbooking.
How many of the above statements are true for you?
I floss everyday.
I have few photos of myself.
I don't procrastinate.
I get down in the dumps for a few days in the row sometimes.
I have stalked people on Facebook.
How many of the above statements are true for you?

Group 3

I drink 6–8 glasses of water a day
I like to cook.
I sleep less than 7 hours per night.
I keep ticket stubs.
I've use Craig's list
How many of the above statements are true for you?
I consider myself a sports fan.
I live in the same state as my parents.
I often make “to-do” lists.
I've had a monogamous relationship.
I enjoy scrapbooking.
How many of the above statements are true for you?
I floss everyday.
I have few photos of myself.
I don't procrastinate.
I get down in the dumps for a few days in the row sometimes.
If I had an STD that was actively being treated, I would alert my current partner
I have stalked people on Facebook.
